# Supplementary material for: Circulating miR-451a Expression May Predict Recurrence in Atrial Fibrillation Patients after Catheter Pulmonary Vein Ablation
Source: Cells. 2023 Feb 16;12(4):638. doi: 10.3390/cells12040638 (PMC9953933; doi:10.3390/cells12040638)
Supplement: Supplementary file 1 [file cells-12-00638-s001.zip › cells-2183305-supplementary.pdf]

Supplemental Table S1. List of microRNAs and miRBase accession number

miRBase Accession #	miRNA ID
MIMAT0000062	hsa-let-7a-5p
MIMAT0000063	hsa-let-7b-5p
MIMAT0000064	hsa-let-7c-5p
MIMAT0000065	hsa-let-7d-5p
MIMAT0000066	hsa-let-7e-5p
MIMAT0000067	hsa-let-7f-5p
MIMAT0000416	hsa-miR-1-3p
MIMAT0000098	hsa-miR-100-5p
MIMAT0000101	hsa-miR-103a-3p
MIMAT0000104	hsa-miR-107
MIMAT0000254	hsa-miR-10b-5p
MIMAT0000421	hsa-miR-122-5p
MIMAT0000422	hsa-miR-124-3p
MIMAT0000443	hsa-miR-125a-5p
MIMAT0000423	hsa-miR-125b-5p
MIMAT0000445	hsa-miR-126-3p
MIMAT0000425	hsa-miR-130a-3p
MIMAT0000427	hsa-miR-133a-3p
MIMAT0000770	hsa-miR-133b
MIMAT0000431	hsa-miR-140-5p
MIMAT0000434	hsa-miR-142-3p
MIMAT0000435	hsa-miR-143-3p
MIMAT0000436	hsa-miR-144-3p
MIMAT0000437	hsa-miR-145-5p
MIMAT0000449	hsa-miR-146a-5p
MIMAT0000450	hsa-miR-149-5p
MIMAT0000451	hsa-miR-150-5p
MIMAT0000646	hsa-miR-155-5p
MIMAT0000417	hsa-miR-15b-5p
MIMAT0000069	hsa-miR-16-5p
MIMAT0000103 MIMAT0000070	hsa-miR-106a-5p hsa-miR-17-5p
MIMAT0000256	hsa-miR-181a-5p
MIMAT0000257	hsa-miR-181b-5p
MIMAT0000259	hsa-miR-182-5p
MIMAT0000261	hsa-miR-183-5p
MIMAT0000455	hsa-miR-185-5p

MIMAT0001412	hsa-miR-18b-5p
MIMAT0000461	hsa-miR-195-5p
MIMAT0000231	hsa-miR-199a-5p
MIMAT0000462	hsa-miR-206
MIMAT0000241	hsa-miR-208a-3p
MIMAT0004960	hsa-miR-208b-3p
MIMAT0000076	hsa-miR-21-5p
MIMAT0000267	hsa-miR-210-3p
MIMAT0000271	hsa-miR-214-3p
MIMAT0000077	hsa-miR-22-3p
MIMAT0000278	hsa-miR-221-3p
MIMAT0000279	hsa-miR-222-3p
MIMAT0000280	hsa-miR-223-3p
MIMAT0000281	hsa-miR-224-5p
MIMAT0000078	hsa-miR-23a-3p
MIMAT0000418	hsa-miR-23b-3p
MIMAT0000080	hsa-miR-24-3p
MIMAT0000081	hsa-miR-25-3p
MIMAT0000082	hsa-miR-26a-5p
MIMAT0000083	hsa-miR-26b-5p
MIMAT0000084	hsa-miR-27a-3p
MIMAT0000419	hsa-miR-27b-3p
MIMAT0000086	hsa-miR-29a-3p
MIMAT0000100	hsa-miR-29b-3p
MIMAT0000681	hsa-miR-29c-3p
MIMAT0000684	hsa-miR-302a-3p
MIMAT0000715	hsa-miR-302b-3p
MIMAT0000087	hsa-miR-30a-5p
MIMAT0000244	hsa-miR-30c-5p
MIMAT0000245	hsa-miR-30d-5p
MIMAT0000692	hsa-miR-30e-5p
MIMAT0000089	hsa-miR-31-5p
MIMAT0000510	hsa-miR-320a
MIMAT0000752	hsa-miR-328-3p
MIMAT0000753	hsa-miR-342-3p
MIMAT0000710 MIMAT0022834	hsa-miR-365a-3p hsa-miR-365b-3p
MIMAT0000732	hsa-miR-378a-3p
MIMAT0001340	hsa-miR-423-3p
MIMAT0001341	hsa-miR-424-5p

MIMAT0001631	hsa-miR-451a
MIMAT0002177	hsa-miR-486-5p
MIMAT0002816	hsa-miR-494-3p
MIMAT0002870	hsa-miR-499a-5p
MIMAT0000252	hsa-miR-7-5p
MIMAT0000092	hsa-miR-92a-3p
MIMAT0000093	hsa-miR-93-5p
MIMAT0000096	hsa-miR-98-5p
MIMAT0000097	hsa-miR-99a-5p
MIMAT0000010	cel-miR-39-3p
MIMAT0000010	cel-miR-39-3p
N/A	SNORD61
N/A	SNORD68
N/A	SNORD72
N/A	SNORD95
N/A	SNORD96A
N/A	RNU6-6P
N/A	miRTC
N/A	miRTC
N/A	PPC
N/A	PPC

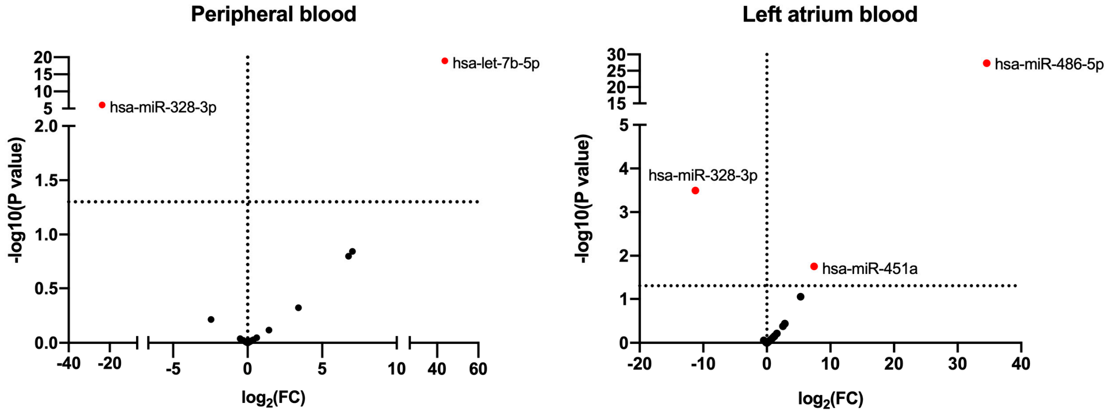

Figure S1. Volcano plots of miRNAs AF patients in plasma from peripheral blood and left atrium blood.

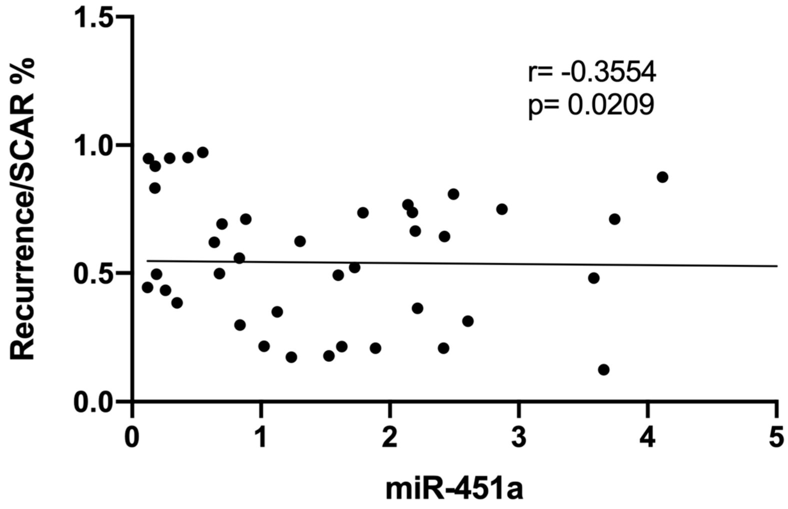

Figure S2. Correlation of miR-451a expression and recurrence/scar size.
